# Clinicopathological Profile of Dermatitis Herpetiformis Patients in Saudi Arabia

**DOI:** 10.7759/cureus.48045

**Published:** 2023-10-31

**Authors:** Bushra Alasmari, Rayan Alkhodair

**Affiliations:** 1 Department of Dermatology, King Abdulaziz Medical City Riyadh, Riyadh, SAU; 2 Medical Research, King Abdullah International Medical Research Center, Riyadh, SAU; 3 Medicine, King Saud bin Abdulaziz University for Health Sciences, Riyadh, SAU; 4 Pediatric Dermatology Division, Department of Pediatrics, King Abdullah Specialized Children’s Hospital, Riyadh, SAU

**Keywords:** immunoglobulin a, direct immunofluorescence, celiac disease, vesiculobullous diseases, dermatitis herpetiformis

## Abstract

Objectives

The aim of this study was to describe the clinical, serological, and histopathological features of patients with dermatitis herpetiformis (DH) in Saudi Arabia.

Methods

We retrospectively reviewed the medical charts of all patients diagnosed with DH in the dermatology departments of National Guard Health Affairs (NGHA) hospitals in five different cities, from 2016 to 2022. We included patients who had been diagnosed by a dermatologist and had a combination of typical DH skin lesions, positive immunoglobulin A (IgA) on direct immunofluorescence (DIF), and/or positive tissue transglutaminase (tTG) IgA.

Results

A total of 11 patients were included. Their average age was 43.6 ± 12.5 years, and the ratio of females: males was 2.7: 1. Among the eight skin biopsies performed, IgA was detected on DIF in five patients. Seven out of nine patients (77.8%) had positive tTG IgA. Nine patients were managed with dapsone and a gluten-free diet (GFD); they had excellent responses within months.

Conclusion

The profiles of Saudi patients with DH were similar to those of Caucasian patients, but DH appears to be less common in Saudi Arabia. The high positive rates of tTG IgA make it an important tool for diagnosis in unclear cases. Dermatitis herpetiformis is likely associated with underlying gluten-sensitive enteropathy in Saudi patients.

## Introduction

Dermatitis herpetiformis (DH) is a chronic, autoimmune, blistering skin condition considered to be an extraintestinal manifestation of celiac disease (CD) [1]. Affected individuals present with intensely itchy polymorphic lesions symmetrically distributed over the forearms, knees, scalp, or buttocks [1]. Dermatitis herpetiformis is characterized by subepidermal blisters and neutrophilic infiltrates at the dermal papillae on histopathology and granular immunoglobulin A (IgA) deposits along the dermal-epidermal junction on perilesional direct immunofluorescence microscopy (DIF) [2]. Nearly all DH patients carry human leukocyte antigen (HLA) haplotypes DQ8 or DQ2, which play a crucial role in promoting an immune response against gliadin peptides and correspond to those found in CD patients [1,3].

Dermatitis herpetiformis is generally rare and most commonly occurs in Caucasian individuals in Europe and North America, where CD is prevalent [4-8]. In addition to the rarity of the disease in Asian populations, a recent study found different features in Japanese patients with DH compared with Caucasian patients. The researchers noted the absence of typical CD serological markers and a different pattern of IgA on DIF [9,10]. The atypical features of DH in Japanese patients may have led to an underestimation of its prevalence in these populations [11]. Similarly, CD was thought to be limited to Caucasian Europeans but is now increasingly found in populations in Northern Africa, the Middle East, India, and Northern China [12,13]. Skin type could be another confounding factor, as various dermatologic conditions have been demonstrated to present differently in individuals with darker skin types [14].

In Saudi Arabia, CD is relatively common, with a seroprevalence comparable to that of countries with high, biopsy-proven CD rates [13]. Despite the accumulating literature on CD in Saudi Arabia, there have been no studies on its cutaneous manifestation, DH. This study aimed to describe the clinical, serological, and histopathological features of patients with DH in tertiary care settings in different regions of Saudi Arabia.

## Materials and methods

This was a cross-sectional retrospective study of patients diagnosed with DH in the dermatology department of National Guard Health Affairs (NGHA) hospitals across five different cities in Saudi Arabia (Riyadh, Jeddah, Al-Ahsa, Dammam, and Medina) from January 2016 to December 2022. NGHA’s electronic healthcare records system “Bestcare” was used for retrieving the data. The study was approved by the local institutional review board. As the study was a retrospective chart review, no informed consent from the patients was required. The study subjects were identified through searching the diagnosis field for “dermatitis herpetiformis.” All identified patients’ medical records were reviewed to ensure the diagnosis. The patients were included in the study only if the diagnosis was made by a dermatologist based on typical findings on DIF of perilesional skin (granular IgA deposits at the papillary dermis) and/or characteristic clinical features (symmetrical, polymorphic lesions such as urticarial papules, plaques, vesicles, blisters, erosions, and excoriations) and positive serological markers (anti-tissue transglutaminase and anti-endomysium IgA).

The following data were collected for each patient: age, gender, age at diagnosis, the morphology of DH lesions, affected anatomical sites, histopathological findings on the skin and small-bowel biopsies, perilesional DIF findings, serological markers of gluten-sensitivity (anti-tissue transglutaminase and anti-endomysium IgA), prior diagnosis of CD, and prior diagnosis of other associated diseases (type 1 diabetes mellitus, atopic dermatitis, vitiligo, alopecia areata, Hashimoto thyroiditis, pernicious anemia, and rheumatoid arthritis). 

The collected data were entered into a Microsoft Excel spreadsheet (Microsoft Corporation, Redmond, WA) and descriptive statistics were used for this study. Means and standard deviation (SD) were used to summarize the continuous variables while frequencies and percentages were used to summarize the categorical variables.

Anti-tissue transglutaminase (anti-tTG) IgA was determined using a Quanta Lite tTG IgA ELISA (Inova Diagnostics, Inc., San Diego, CA, USA). tTG IgA value >30 units were interpreted as moderate to strong positive, 20-30 as weak positive, and <20 units as negative. Anti-endomysium (anti-EMA) IgA titers >1:10 were interpreted as positive and titers <1:10 as negative.

## Results

Demographics and clinical features

We found 11 patients with DH during the study period (Table 1). The female-to-male ratio was 2.7:1. Patients’ ages ranged between 19 and 59, with a mean of 43.6 ± 12.5 years, and the average age of onset was 40.1 ± 11.2 years. Five patients (45.5%) were in Riyadh, five (45.5%) were in Jeddah, and one (9%) was in Al-Ahsa. Most patients experienced symptoms for over a year before consulting a dermatologist. The skin lesions were polymorphic, manifesting as vesicles, bullae, urticarial plaques, or excoriated papules. Additionally, all patients complained of severe itching that accompanied the rash. The most commonly affected sites were the elbows and knees, followed by the back and buttocks. Four patients had lesions at unusual sites, such as the face and palms, as well as at the common sites like the elbows and knees. Associated autoimmune diseases were only found in three patients; two had rheumatoid arthritis and one had hypothyroidism. Skin biopsies were performed in 8 out of 11 patients. Two of the three patients who were not biopsied had already been diagnosed elsewhere and had been referred to us for continuity of disease management while one did not have active skin lesions at the time of presentation.

**Table 1:** Overview of 11 patients with dermatitis herpetiformis *A positive result was defined as >20 units for tTG IgA and a titer of > 1:10 for endomysium IgA; †Typical DH biopsies were defined as those with positive IgA on DIF; ‡Based on duodenal biopsy: four patients had a prior diagnosis of CD with overt gastrointestinal symptoms while one patient had CD features on duodenal biopsy after rash appearance tTG: tissue-transglutaminase; DH: dermatitis herpetiformis; GFD: gluten-free diet; GSE: gluten-sensitive enteropathy

Patients with dermatitis herpetiformis		
	N / N of available data	%
Gender		
Male	3/11	27.3%
Female	8/11	72.7%
Age (Mean ± SD) (years)	43.6 ± 12.5	
Age at onset (Mean ± SD) (years)	40.1 ± 11.2	
Site of lesions		
Elbows	9/10	90%
Knees	9/10	90%
Buttocks	2/10	20%
Back	3/10	30%
Abdomen	1/10	10%
Face	1/10	10%
Palms	3/10	30%
Positive serology*		
tTG IgA	7/9	77.8%
Endomysium IgA	2/3	66.7%
Skin biopsy†		
Typical DH	5/8	62.5%
Atypical	3/8	37.5%
Gluten-sensitive enteropathy‡	5/11	45.5%
Associated autoimmune diseases		
Rheumatoid arthritis	2/11	18.2%
Hypothyroidism	1/11	9.1%
Therapy		
Dapsone + GFD	9/11	81.8%
Topical steroids	2/11	18.2%

Histopathology

Among the eight patients who had skin biopsies, five had subepidermal blisters with neutrophilic infiltrates. Three patients had inconclusive histopathology results, with two exhibiting subepidermal edema and one exhibiting subepidermal fibrosis; all three patients exhibited mixed cellular infiltrates comprising perivascular lymphocytes and scattered interstitial eosinophils.

DIF

Direct immunofluorescence was performed on perilesional skin biopsies in eight patients. Granular IgA deposits were detected in five patients; in two patients, they were at the tips of the papillary dermis and in three patients, they were within the dermal papillae. In one patient, granular IgA deposition occurred at the dermo-epidermal junction with accentuation at the tips of the papillary dermis. Granular IgA and granular C3 were found in the basement membrane and dermal papillae in another patient. DIF was negative for IgA, IgG, IgM, and C3 in three patients, in whom DH was strongly suspected and had symptomatic improvement when dapsone and a gluten-free diet (GFD) were prescribed.

Gluten-sensitive enteropathy (GSE)

Seven patients were positive for tTG IgA, and four of these patients had been diagnosed with CD prior to rash onset. One patient was suspected to have DH but only had post-inflammatory hyperpigmentation (PIH) at presentation; thus, a skin biopsy was not performed. The patient was positive for tTG IgA and exhibited features of CD on duodenal biopsy, despite being completely asymptomatic. The remaining two patients did not undergo duodenal biopsies and had no gastrointestinal complaints. Endomysium IgA was only performed for three patients; two patients tested positive and these same patients were also positive for tTG IgA.

Treatment

Once the diagnosis was confirmed, nine patients were advised to follow a strict GFD and were started on dapsone at an initial dose of 25 mg, which was gradually increased to 100 mg. All except one patient attended subsequent follow-up visits and showed significant improvement within less than six months of treatment. Dapsone was well-tolerated by most patients; however, one patient developed abnormal liver function tests and high reticulocytes after being maintained on a high dose of dapsone (100-200 mg) for a year. Another patient developed an episode of dark urine with elevated reticulocytes, which resolved after dose adjustment. Two patients were treated with topical steroids while awaiting biopsy results and did not attend follow-up visits.

## Discussion

Dermatitis herpetiformis is a rare disease most commonly reported in Western countries although reports from Eastern and Southeast Asia are increasing [9,15,16]. Our study provided a description of the clinicopathological profile of DH patients in Saudi Arabia. We found 11 patients who fit the criteria for DH during the study period. In the USA and Europe, the reported prevalence of DH ranged from 11.2 to 75.3 per 100,000, whereas the prevalence in Asian populations seems to be much lower [11,16]. This disparity has been attributed to the rarity of CD, comparatively lower wheat consumption in Asia, and the lack of HLA types predisposing one to DH in these populations [11]. Despite the prevalence of CD in Saudi Arabia, our results suggest that DH is uncommon [17]. This could be due to the substantially decreasing global incidence of DH after 1990, which is hypothesized to be a result of improved subclinical CD detection [7,18].

In accordance with previous studies, the average age of onset was in the fifth decade [15,16]. There was an unexplained predominance of DH in females; they accounted for 72.7% of the study subjects, which contradicts the male preponderance reported in the literature [11,15,16]. Although most skin lesions were at common sites, unusual sites, such as the palms and face, were also affected in a few patients. Acral petechiae have been described as a rare manifestation of DH [19,20]. However, we found one patient who initially only had itchy urticarial plaques over their palms with a history of similar lesions over the knees and elbows, which showed as PIH on physical examination. The patient was later proven to have positive serology, CD features on duodenal biopsy, and their skin improved with dapsone and a GFD. Dermatitis herpetiformis is typically suspected in patients with pruritic vesicles over the extensor surfaces of the extremities [11]. The differential diagnosis of DH includes a wide range of other bullous and pruritic disorders [11]. As such, it is important to be aware of atypical presentations to avoid misdiagnosis and treatment delay.

Highly sensitive and specific, perilesional DIF is considered the gold-standard test to confirm a DH diagnosis [11]. In our study, all five patients with positive DIF had granular IgA at the dermal papillae, the pathognomonic pattern for DH. However, three patients had negative DIF by perilesional skin biopsy despite having other features suggestive of DH, including positive serology, typical clinical features, and responsiveness to dapsone and a GFD. This occurrence has been described by several authors in the literature, with some patients demonstrating positive DIF upon subsequent skin biopsies [11,15]. Recent guidelines published by the European Society for the Study of Coeliac Disease recommended serology testing and HLA DQ testing to ascertain the diagnosis of DH in patients with negative IgA on DIF [21].

Early studies from Western countries have established the CD-specific tTG IgA and endomysium IgA antibodies as essential tests to aid in the diagnosis of DH [11]. However, recent reports on East Asian populations found these antibodies less helpful, with only 38% of DH patients having positive tTG IgA [11]. In contrast, our results showed tTG to be positive in 77% of patients, which is comparable to the seropositive rate of 72.5% reported in Caucasian DH patients [11]. A plausible explanation for this finding is the high prevalence of HLA DQ8 and DQ2 and the excessive consumption of gluten-rich foods in our population, which are also features of Western DH patients [11,22]. Although epidermal transglutaminase (eTG) IgA is more sensitive and specific for DH, kits for eTG IgA are still not readily available at most institutions [11]. Therefore, we believe tTG IgA could be a valuable test for DH patients in our population, especially in those with equivocal skin biopsy results.

The mainstay of DH management is strict adherence to a GFD [11]. Most of our patients significantly improved on dapsone and a GFD, and lesions were completely resolved in four patients. These patients were not weaned off dapsone but maintained on a low dose of 25 mg. Two patients had evidence of hemolysis on laboratory tests while on high doses of dapsone, which resolved upon dose reduction. Several patients reported flare-ups after gluten ingestion, further reinforcing the relationship between GSE and DH found in Caucasian DH patients but not in Japanese patients [9]. Due to the potential side effects and careful monitoring dapsone requires, efforts should be made to ensure strict adherence to GFD and to taper the medication off whenever possible [23].

A main limitation of our study was the small sample size, which limits the generalizability of the results to all DH patients. Additionally, we retrospectively reviewed medical records that were not specifically intended for data collection; thus, some information was missing. Although more extensive studies would reflect the prevalence and clinical features more accurately, our study provides insight into the characteristics of some Saudi patients with DH.

## Conclusions

In conclusion, the clinical and pathological characteristics of 11 patients with DH across Saudi Arabia aligned with those of Caucasian DH patients. The positive rate of tTG IgA was similar to that of western DH patients, making it an essential investigation in our population. Unlike Japanese patients, there appears to be an association between GSE and DH in Saudi patients. Almost all patients had a satisfactory response to dapsone and a GFD.
