# Correction: Clinicopathological Profile of Dermatitis Herpetiformis Patients in Saudi Arabia

**DOI:** 10.7759/cureus.c150

**Published:** 2024-01-03

**Authors:** Bushra Alasmari, Rayan Alkhodair

**Affiliations:** 1 Department of Dermatology, King Abdulaziz Medical City Riyadh, Riyadh, SAU; 2 Medical Research, King Abdullah International Medical Research Center, Riyadh, SAU; 3 Medicine, King Saud bin Abdulaziz University for Health Sciences, Riyadh, SAU; 4 Pediatric Dermatology Division, Department of Pediatrics, King Abdullah Specialized Children’s Hospital, Riyadh, SAU

This article has been corrected to accurately reflect the affiliations of the authors. The following affiliations have been added: 

Bushra Alasmari Medical Research, King Abdullah International Medical Research Center, Riyadh, SAU

Rayan Alkhodair Medicine, King Saud bin Abdulaziz University for Health Sciences, Riyadh, SAU Medical Research, King Abdullah International Medical Research Center, Riyadh, SAU

The authors deeply regret that these errors were not identified and addressed prior to publication.

 

